# High genetic and epigenetic variation of transposable elements: Potential drivers to rapid adaptive evolution for the noxious invasive weed *Mikania micrantha*

**DOI:** 10.1002/ece3.8075

**Published:** 2021-09-15

**Authors:** Yingjuan Su, Qiqi Huang, Zhen Wang, Ting Wang

**Affiliations:** ^1^ School of Life Sciences Sun Yat‐sen University Guangzhou China; ^2^ Research Institute of Sun Yat‐sen University Shenzhen China; ^3^ College of Life Sciences South China Agricultural University Guangzhou China

**Keywords:** adaptive evolution, genetic and epigenetic variation, genetic paradox, *Mikania micrantha*, transposable elements

## Abstract

Why invasive species can rapidly adapt to novel environments is a puzzling question known as the genetic paradox of invasive species. This paradox is explainable in terms of transposable elements (TEs) activity, which are theorized to be powerful mutational forces to create genetic variation. *Mikania micrantha*, a noxious invasive weed, in this sense provides an excellent opportunity to test the explanation. The genetic and epigenetic variation of 21 invasive populations of *M. micrantha* in southern China have been examined by using transposon display (TD) and transposon methylation display (TMD) techniques to survey 12 TE superfamilies. Our results showed that *M. micrantha* populations maintained an almost equally high level of TE‐based genetic and epigenetic variation and they have been differentiated into subpopulations genetically and epigenetically. A similar positive spatial genetic and epigenetic structure pattern was observed within 300 m. Six and seven TE superfamilies presented significant genetic and epigenetic isolation by distance (IBD) pattern. In total, 59 genetic and 86 epigenetic adaptive TE loci were identified. Of them, 51 genetic and 44 epigenetic loci were found to correlate with 25 environmental variables (including precipitation, temperature, vegetation coverage, and soil metals). Twenty‐five transposon‐inserted genes were sequenced and homology‐based annotated, which are found to be involved in a variety of molecular and cellular functions. Our research consolidates the importance of TE‐associated genetic and epigenetic variation in the rapid adaptation and invasion of *M. micrantha*.

## Graphical abstract

*Mikania micrantha* populations maintained an almost equally high level of TE‐based genetic and epigenetic variation and they have not been differentiated into subpopulations genetically and epigenetically. 51 genetic and 44 epigenetic loci were found to correlate with 27 environmental variables (including longitude, latitude, precipitation, temperature, vegetation coverage, and soil metals). Twenty‐five transposon‐inserted genes were sequenced and homology‐based annotated, which are found to be involved in a variety of molecular and cellular functions.

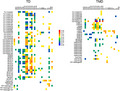

## 1 INTRODUCTION

Why invasive species can rapidly adapt to novel environments has been a puzzling issue for decades (Stapley et al., 2015). Not all introduced populations undergo genetic bottlenecks. In cases if the populations have experienced genetic bottlenecks, they are assumed to have low genetic diversity and low evolutionary potential. However, often bottleneck populations retain the ability to successfully colonize new ranges, forming a phenomenon known as the genetic paradox in invasive species (Frankham, 2005). Several possible explanations have been proposed for this paradox: admixture (Ferrero et al., 2015), intraspecific hybridization (Hohenlohe et al., 2013), unaffected quantitative trait variation (Taft & Roff, 2012), and the existence of mutational processes to generate novel genetic variation (Casacuberta & González, 2013). More recently, transposable elements (TEs) are invoked to explain the paradox as they may act as powerful mutational forces to create genetic variation for rapid adaptation (Stapley et al., 2015).

TEs represent a dominant feature of angiosperm genomes because they often constitute the major fraction of DNA (Oliver et al., 2013). As a dynamic reservoir of sequence variation, TEs impose profound effects on host genomes through regulating gene expression, genomic rearrangements, mutations, epigenetic variation, and phenotypic variation (Böhne et al., 2008; Gabriel et al., 2006). The elements are mainly classified into two classes: retrotransposons (class I) and DNA transposons (class II) (Stapley et al., 2015). The former have, while the latter lack, an RNA transposition intermediate. Class I is further divided into two subclasses: LTR (long terminal repeats) and non‐LTR retrotransposons (Schrader & Schmitz, 2019). Class II can also be grouped into two subclasses based on the number of DNA stands that are cut during transposition. TEs, as mobile genetic units called “jumping genes”, trigger a broad range of molecular mutations in a population including newly created genetic and epigenetic variation. Stapley et al. (2015) hypothesize that TE activity enables to generate potentially beneficial alleles at a rate higher than the background rate of mutation. As a result, populations may have a more rapid adaptation to environmental fluctuations by maintaining TE activity than resorting to other mutational input (Casacuberta & González, 2013; Schrader & Schmitz, 2019). Up to now there are several reports on the roles of TEs in adaptation and phenotypic diversification in species such as *Cardiocondyla obscurior* (Schrader et al., 2014), *Aedes albopictus* (Goubert et al., 2017), and *Capsella rubella* (Niu et al., 2019). Nevertheless, no TE‐related studies have been described for invasive weeds. In this context, *Mikania micrantha* offers an opportunity to explore the causal link between genetic and epigenetic variation of TEs and invasion mechanisms.

Transposon display (TD) and transposon methylation display (TMD) are powerful techniques to investigate TE genetic and epigenetic variation, respectively. TD is capable to identify the integration site of transposons in gene tagging (Takagi et al., 2003), whereas TMD surveys CCGG methylation in DNA on the flanks of transposons by applying two CCGG methylation‐sensitive isoschizomers *MspI* and *HpaII* (Kashkush & Khasdan, 2007; Yaakov & Kashkush, 2011). *Ms* *pI* is sensitive only when the external cytosine is methylated, whereas *HpaII* is sensitive to methylation of either cytosine (except when the external cytosine is hemi‐methylated) (Kashkush & Khasdan, 2007; Yaakov & Kashkush, 2011). A combined use of the two enzymes leads to the detection of cytosine methylation status. TD and TMD share similar experimental methods except for adaptors, primers, and the restriction enzyme.

In southern China, *M. micrantha* has been rapidly expanding since being introduced in 1984 (Zhang et al., 2010). Previous studies have noted the association of multiple environmental factors with the invasion of *M. micrantha*: (a) temperature and precipitation changes have effects on growth and reproduction mode; (b) the weed shows strong endurance to heavy metals; and (c) it appears sensitive to light environment change caused by vegetation coverage (Fu et al., 2015; Wang et al., 2012; Zhang et al., 2011). However, the (epi) genetic mechanisms of *M. micrantha* invasion remain elusive. Of note, the invasive populations of *M. micrantha* maintain high levels of genetic diversity as raw materials for evolution, although they have undergone severe genetic bottlenecks during the invasion process (Wang et al., 2008, 2012; Yang et al., 2017). So far, a plausible explanation for this genetic paradox is lacking in *M. micrantha*.

In this study, we have employed TD and TMD to investigate the genetic and epigenetic variation of TEs in the *M. micrantha* populations in southern China. Twenty‐one populations from six different regions have been sampled to cover all areas as possible. The goal is to reveal the relative contribution of TE genetic and epigenetic variation to the rapid adaptation of *M. micrantha*, identify functional genes affected by TE insertions, and characterize TE‐associated adaptive loci and their association with environmental variables.

## 2 MATERIALS AND METHODS

### 2.1 Sample collection and DNA extraction

**FIGURE 1:**
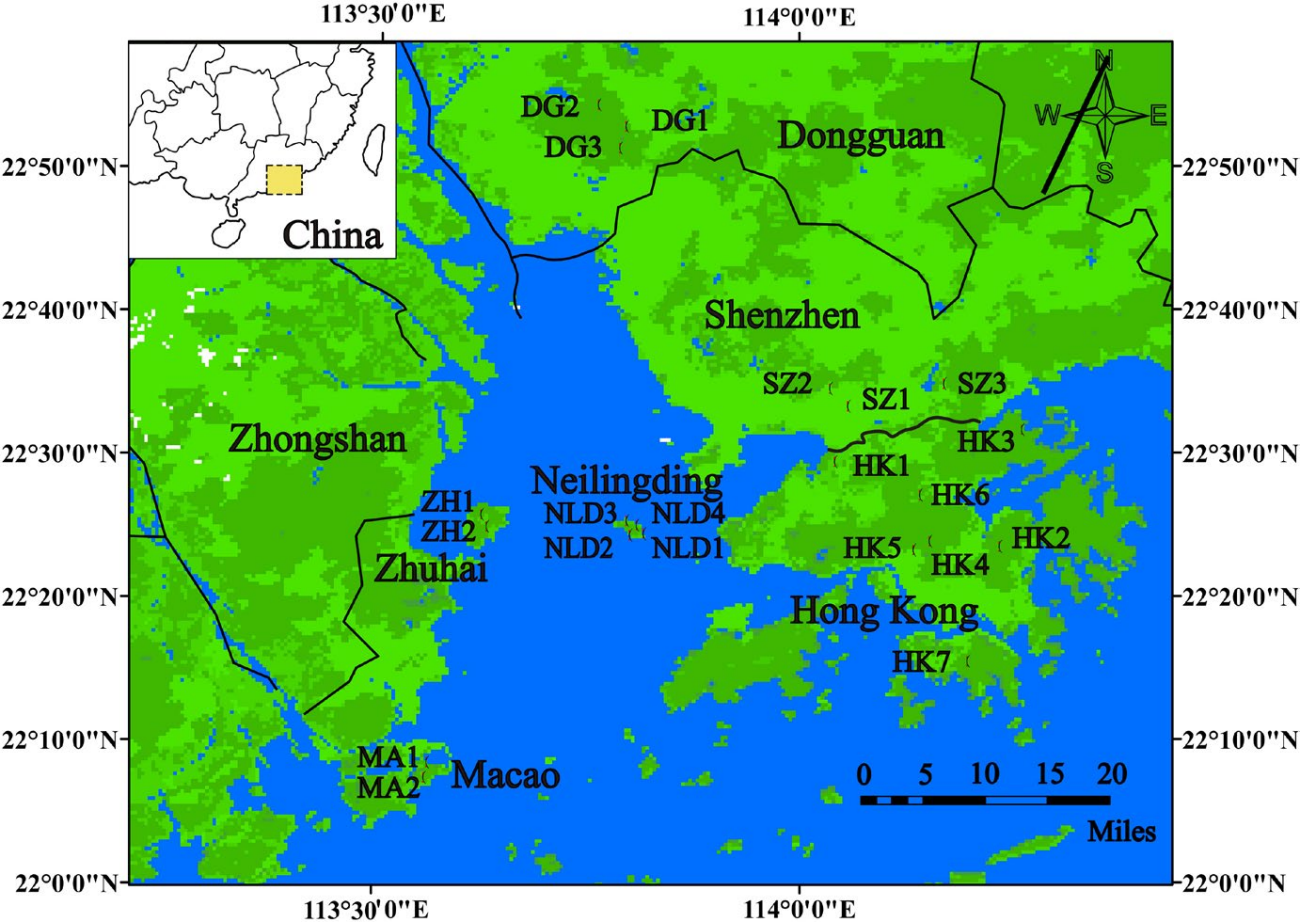
Sampling sites of *Mikania micrantha* in southern China. The red dots show its occurrence location, which are distributed in six regions including Hong Kong, Shenzhen, Neilingding, Zhuhai, Macao, and Dongguan. See Table S1 for population codes

*Mikania micrantha* H.B.K. (Asteraceae) is listed as one of the top ten worst weeds (Holm et al., 1977; Lowe et al., 2000). It is a multi‐branched, perennial, scrambling vine native to Central and South America (Holm et al., 1977) and has become highly invasive in southeast Asia and southern China (Bravo‐Monzón et al., 2018; Wang et al., 2012; Waterhouse, 1994). It performs efficient sexual reproduction by producing enormous numbers of light‐weighted seeds that are dispersed by wind, water, and animals. Moreover, it can also reproduce vegetatively via ramets generated from stem fragments (Clements et al., 2019). *Mikania micrantha* has caused huge damage to agriculture, forestry, and ecological system across its invaded range (Zhang et al., 2010). In total, 306 *M. micrantha* individuals were sampled from 21 populations in six regions (Figure 1 and Table S1), covering majority of its range in southern China. In each population, we randomly collected fresh leaves from 10 to 16 individuals and preserved them in silica gel. Genomic DNA was extracted following the modified CTAB protocol (Su et al., 2005).

### 2.2 Selection of transposable elements

Based on genome sequence of *Helianthus annuus* (Badouin et al., 2017; Gill et al., 2014; Giordani et al., 2014; Staton et al., 2012) and horizontal transferability of transposons (Du et al., 2013; Flavell et al., 1992; Yaakov et al., 2013), we screened active transposons and obtained relative primers (Table 1). Additionally, in order to fully investigate genetic and epigenetic variation, different TE classes were selected to cover as many transposons as possible (Table 1). Firstly, six retro‐TE superfamilies were adopted, including Copia‐like and Gypsy‐like long terminal repeat (LTR) elements, long interspersed elements (LINEs), short interspersed elements (SINEs), LARD, and TRIM. Secondly, six DNA‐TE superfamilies were employed including CACTA, Harbinger, Helitron, Mariner‐like elements, MUDER, and miniature inverted‐repeat transposable elements (MITEs).

**TABLE 1:** Information of TE superfamily, primer name and sequence, and TD *Mse* I and TMD H/M primer

TE superfamily	Primer name	Primer sequence	TD *Mse* I primer	TMD H/M primer	Reference
Ty1‐Copia	*Tnt1‐OL16*	TTCCCACCTCACTACAATATCGC	Mse‐1	HM‐1	Paz et al. (2015)
	*Tto1‐6*	CACTCCCCTGTTAGGAAACATTC	Mse‐1	HM‐1	Paz et al. (2015)
	*Copia35*	TCGATGCAGGGAATGATAAG	Mse‐3	HM‐1	Yadav et al. (2015)
	*Copia825*	ACAACCGATCAGAAGGCAAA	Mse‐2	HM‐1	Yadav et al. (2015)
	*copia300*	ACGGCGTAGCGTTTGATAGC	Mse‐3	HM‐1	Yadav et al. (2015)
	*Solo_Copia_1584*	TAACCTTTGGGCTTGTGGAA	Mse‐1	HM‐1	Yadav et al. (2015)
Ty3‐Gypsy	*gypsy1033*	TCATTCTCAATCAGAGAGGGG	Mse‐2	HM‐1	Yadav et al. (2015)
	*gypsy101*	TCACAGCATGCTTGTTGACA	Mse‐1	HM‐1	Yadav et al. (2015)
	*Gypsy940*	ATCCGATGCAGGGAACGATA	Mse‐2	HM‐1	Yadav et al. (2015)
	*Gypsy358*	GACGACGGTTCAGGTTTGTT	Mse‐2	HM‐2	Yadav et al. (2015)
	*Gypsy433*	TGAGGCCCTTCATAGGATTG	Mse‐1	HM‐1	Yadav et al. (2015)
	*gypsy1010*	TACGCCGGAACAGGGTAGT	Mse‐3	HM‐1	Yadav et al. (2015)
LARD	*Sukkula9900*	GATAGGGTCGCATCTTGGGCGTGAC	Mse‐2	HM‐1	Hysing et al. (2008)
TRIM	*Veju*	GACGGTATGCCTCGGATTTA	Mse‐2	HM‐1	Venetsky et al. (2015)
LINE	*LINE2650*	TTGCACGGAAGAAATGGACA	Mse‐2	HM‐1	Yadav et al. (2015)
	*RJMT*	TGATCATGCTTCTCCACACC	Mse‐2	HM‐1	Yadav et al. (2015)
SINE	*T56*	ATGGATGATGTGCATCGAGA	Mse‐3	HM‐1	Liu (2014)
	*Au SINE R*	GGGAAGGGTCCGACCACTT	Mse‐2	HM‐1	Yaakov et al. (2013)
MITE	*Aison*	TTAAATCGTCAACCTAGAACTACGC	Mse‐1	HM‐2	Yaakov et al. (2013)
	*Eos*	CAGGGGTGCTTGGAACTTTA	Mse‐2	HM‐2	Yaakov et al. (2013)
	*Oleus*	CAAATCAGAAAGCTGGAACATC	Mse‐1	HM‐2	Yaakov et al. (2013)
	*Minos*	GTCAAAATTGAAGCACGTGGA	Mse‐1	HM‐2	Domb et al. (2017)
	*Tantalos*	TCTAGACAAACCTAGTATGCGGAGT	Mse‐2	HM‐2	Domb et al. (2017)
	*mPing 1*	GCTGACGAGTTTCACCAGGATG	Mse‐2	HM‐2	Cui (2012)
MUDR	*N39‐MUDR*	TTGGCGTACTCCTCTCCTCG	Mse‐2	HM‐1	Lilia (2015)
Tc1‐Mariner	*T67‐Tc1 Mariner*	TCGCTGCAGTATTGTGATCC	Mse‐2	HM‐1	Liu
Helitron	*Cd17*	GCAGTACAGGAGACTCGTA	Mse‐2	HM‐1	Lai et al. (2005)
	*2ph8*	TACAGGCACGCAGGAGCGTAGAA	Mse‐1	HM‐2	Lai et al. (2005)
	*N‐Hel‐IT1*	TGTGGCTTTTTTATATAGTAAGAT	Mse‐1	HM‐1	Choi et al. (2007)
	*5′‐Hel‐It*	GGCTCCAATGTGGTTCCCAA	Mse‐3	HM‐1	Choi et al. (2007)
CACTA	*Balduin*	CAGCTAGCAGACAACAAGGA	Mse‐1	HM‐1	Yaakov and Kashkush (2011)
	*dna13398*	CCCTGTGATCGCAACATCTTT	Mse‐2	HM‐1	Yadav et al. (2015)
PIF‐Harbinger	*T100*	CGTGCTTTTGGAGTCTTGC	Mse‐1	HM‐1	Liu (2014)
	*Dna16027*	CTGCTTCTCCACGTGCTTAA	Mse‐1	HM‐1	Yadav et al. (2015)

### 2.3 Transposon methylation display and transposon display

We used a modified TMD protocol as previously described (Kashkush & Khasdan, 2007; Yaakov & Kashkush, 2011). After digested with either *HpaII* or *MspI*, fragments were ligated with *MseI*/*HpaII*‐*MspI* and *EcoRI* adaptors. *Mse*I‐adaptor: (+) 5′‐TACTCAGGACTCAT‐3′ (−) 3′‐GAGTCCTGAGTAGCAG‐5′. *Msp*I/*Hpa*II‐adaptor: (+) 5′‐GATCATGAGTCCTGCT‐3′ (−) 3′‐AGTACTCAGGACGAGC‐5′. *Eco*RI‐adaptor: (+) 5′‐CTCGTAGACTGCGTACC‐3′ (−) 3′‐CATCTGACGCATGGTTAA‐5′.

Pre‐amplification PCR was conducted with one TE‐specific primer and another primer complimentary to the core sequence of the *HpaII*/*MspI* adaptor. The pre‐amplification was performed in 20 μl total reaction volumes, including 5 μl of template DNA, 3.5 μl of 10× PCR buffer, 1.5 μl of dNTPs (2.5 mM each), 0.5 μl of each primer (10 μM), and one unit Taq DNA polymerase. The PCR conditions were as follows: initial denaturation at 94℃ for 30 s; followed by 23 cycles at 94℃ for 30 s, 56℃ for 30 s, and 72℃ for 1 min; and a final extension step at 72℃ for 10 min. Monomorphic bands in both *HpaII* and *MspI* digestions were regarded as nonmethylated CCGG sites, whereas presence–absence matrices were used to accommodate methylated types.

Similar to the above pre‐amplification, selective amplification was performed to acquire different chimeric sequences (TE/flanking DNA) by using various combinations of adaptor primers with different selective bases and a specific 5‐FAM end‐labeled TE primer. The selective PCR conditions were as follows: initial denaturation at 94℃ for 30 s; 15 cycles of 94℃ for 30 s, 66.5℃ for 30 s (decreasing 0.7℃ per cycle), and 72℃ for 1 min; followed by 23 cycles of 94℃ for 30 s, 56℃ for 30 s, and 72℃ for 1 min; and a final extension step at 72℃ for 10 min. The PCR products were electrophoresed in an ABI 3730 DNA analyzer (Applied Biosystems) with the GeneScan 1200 LIZ Internal Size Standard (Applied Biosystems).

Transposon display was achieved using the methylation‐insensitive restriction enzyme *MseI*, rather than *HpaII* and *MspI* used in TMD. The *MseI*‐adaptor pair and the TE‐specific primer were shown in Table 1 and Table S2.

Transposon methylation display and TD bands were extracted and reamplified (using the same PCR conditions as in the selective amplification reaction) and sequenced. Respective restriction enzymes, adaptors, and primers of TMD and TD were listed in Table 1 due to their similar protocols.

### 2.4 Soil composition analysis

Soil samples collected from 21 *M. micrantha* invasive populations were air‐dried and ground to pass through a sieve with an aperture size of 1 mm and 0.2 mm. Soil moisture was determined by oven‐drying for 6 hr at 105℃. The measurement of soil pH and electrical conductivity was performed in a solution of soil mixed with water at a ratio of 1:5 (W:V) using a pH meter (DPS‐307A, INESA, Shanghai, China) and a conductivity meter (DPS‐307A, INESA), respectively. Soil organic matter content was measured by using the potassium dichromate volumetry method. Soil total nitrogen was quantified by Kjeldahl method using KjeltecTM 8400 Analyzer Unit (Foss) after digestion at a ratio of 1:10 (W:V) soil to H_2_SO4. Total carbon was determined in a Total Organic Carbon Analyzer (Shimadzu) under the burning conditions of 720℃. Soil K, Ca, Na, Mg, Al, P, S, Si, Fe, Mn, Zn, Cu, Pb, Cr, As, Se, Ni, and Cd were assayed using an Inductively Coupled Plasma Optical Emission Spectrometer (ICP‐OES, PerkinElmer) after acid digestion. Experimental treatments without soil samples were served as control. All the measurements were repeated three times.

### 2.5 Data analysis

GeneMarker v2.2 (Hulce et al., 2011) was used to analyze raw fluorescent TD and TMD data. Two marker profiles were scored for the presence (1) or absence (0) of bands, and a binary matrix was generated for each TE superfamily.

GenAlEx v6.5 (Peakall & Smouse, 2012) was used to calculate genetic and epigenetic parameters, including number of loci, percentage of polymorphic loci (*%P*), Shannon information index (*I*), and unbiased expected heterozygosity (*uHe*), as well as percentage of permethylation (*%Per*), hemimethylation (*%Hemi*), and nonmethylation (*%Non*).

We performed analysis of molecular variance (AMOVA) through Arlequin v3.0 with 1,000 random permutations (Excoffier & Lischer, 2010). For each TE superfamily, three levels were partitioned: among regions, among populations, and within population. Mantel and partial mantel tests were carried out to investigate the correlation among populations between genetic and epigenetic differentiation, and genetic or epigenetic differentiation and geographic distances (km) with 100,000 permutations. The matrix of genetic and epigenetic differentiation contained pairwise *Fst* values.

Bottleneck v1.2.02 (Cristescu et al., 2010) was used to investigate population bottleneck effect based on heterozygosity excess. We adopted the sign test under the infinite allele model (I.A.M.) and the stepwise mutation model (S.M.M.) with 1,000 replications to compute the distribution of gene diversity expected from the observed number of alleles, given sample size under the assumption of mutation‐drift equilibrium. Bottleneck signatures were identified by a heterozygosity excess/deficiency ratio (*He*/*Hd*) that significantly deviated from the expected ratio (1:1) at mutation–drift equilibrium (*p* < .05).

To detect linkage disequilibrium (LD) between two loci, we implemented TASSEL v3.0 (Bradbury et al., 2007) for each TE superfamily. LD was identified based on the correlation coefficient (*r* ^2^) (*r* ^2^ > 0.3, *p* < 0.001).

The Bayesian clustering method was used to investigate the genetic structure of populations implemented in STRUCTURE v2.2 (Pritchard et al., 2000). The likelihood values across multiple values of *K* (1–24) were assessed based on Structure Harvester (http://taylor0.biology.ucla.edu/structureHarvester/) (Earl & vonHoldt, 2012). The graphics were obtained and visualized with Distruct v1.1 (Rosenberg, 2004), and Clumpp v1.1.2 (Jakobsson & Rosenberg, 2007) was used to align the ten replicates for *K* with 100,000 MCMC interactions and a 10,000 burn‐in period. In addition, PAST v3.19 was used to perform principal component analysis (PCA) to further find the eigenvalues and eigenvectors of population correlation matrix in a multidimensional dataset (Hammer et al., 2001). Parameter set included as follows: Recompute: Matrix = variance–covariance; Groups = between‐group; Bootstrap *N* = 9,999.

To survey spatial genetic structure (SGS), we used Moran's *I* value in SAM v4.0 to analyze the spatial autocorrelation of 21 populations (Rangel et al., 2010). As the spatial autocorrelation measure, Moran's *I* value usually ranges from −1 to +1. The former shows negative spatial autocorrelation, while the latter indicates positive spatial autocorrelation. Based on the maximum distance between two populations (89 km), nine distance intervals were set: 0–10, 10–20, 20–30, 30–40, 40–50, 50–60, 60–70, 70–80, and 80–90 km. The upper and lower 95% confidence intervals and level of significance (*P*) were established for each observed value and each distance class using 9,999 Monte Carlo permutations of individuals among different distance classes. SPAGeDi v1.3d was used to further assess fine‐scale spatial genetic structure through estimating *Sp* statistic based on *Sp* = −*b_k_*/(1 − *θ*_1_) (*θ*_1_: average coancestry coefficient; *b_k_*: slope of the regression curve) (Hardy & Vekemans, 2002). Seven fine distance intervals were set: intra‐group, 0–0.15, 0.15–0.30, 0.30–0.45, 0.45–0.60, 0.60–0.75, and 0.75–0.90 km. We also calculated kinship coefficients (*F_ij_*) between two pair populations, the significance of which was estimated through 9,999 permutations.

Signatures of selection and outlier detection were carried out by using BayeScan (Foll & Gaggiotti, 2008) and Dfdist (http://www.rubic.rdg.ac.uk/~mab/stuff), a modification for dominant markers of the software developed by Beaumont and Nichols (1996). The two different outlier detection approaches were simultaneously performed to ensure the accuracy of results. Based on the multinomial‐Dirichlet model, BayeScan can identify selective loci, using a reversible‐jump MCMC algorithm to calculate the posterior probability of the models, compare two alternative models with and without selection, and estimate the departure from neutrality at a given locus (Foll & Gaggiotti, 2008). In comparison with other methods, the Bayesian model‐based approach is very powerful due to directly controlling the false discovery rate (FDR). The analyses were run with parameters as iterations = 5,000; pilot runs = 20; burn‐in = 50,000; sample size = 5,000; thining interval = 10; and a locus was regarded as outlier under strong selection if log_10_ (posterior odds, PO) was ≥2. As a widely used frequentist method based on a symmetrical island model, Dfdist enables to detect signatures of selection by comparing observed *F_ST_* of each locus to coalescent‐simulated neutral global distributions by excluding 30% of the highest and lowest *F_ST_* values. *F_ST_* values above the upper 99.5% quantile were considered as outliers. The graphic was obtained using R v3.2.5 (R Core Team, 2013). Parameters of the four modules in the program (Ddatacal, Dfdist, cplot2, and pv2) were set according to Caballero et al. (2008).

Samβada v0.4.5 (Stucki et al., 2017) was used to investigate the associations between signatures of selection and bioclimatic environmental variables. Based on logistic regression models, the software can determine the probability of allele presence/absence for a specific genetic marker in a given environment (Cesconeto et al., 2017). Significance is assessed with both log‐likelihood ratio (*G*) and Wald tests with a Bonferroni correction for multiple comparisons (Joost et al., 2007). The model was considered as fit only when the *G* and Wald scores were significant, with Bonferroni correction at a 99% confidence. Associations between the loci and environmental variables were further determined based on corrected *p*‐values (<1 × 10^–6^). Based on the above results, we further applied the univariate linear regression model in SAM v4.0 to estimate the correlation (*r* ^2^) between outliers and environmental variables (Rangel et al., 2010). Only for *r* ^2^ ≥ 0.5, the associations were regarded as significant. The environmental variables included 19 climate factors (download from WorldClim v.1.4, http://www.worldclim.org/), ten ecological factors (LPDAAC, http://lpdaac.usgs.gov), and 24 soil factors (Appendix S1 and S2; Shen et al., 2021). Variance inflation factor (VIF) was used to measure the correlation between predictor variables. The variables with VIF below five were selected for analysis by runing *vifstep* function of the R package *usdm* (Naimi et al., 2014).

For sequences flanking TEs, we searched against Nr (NCBI non‐redundant database, https://blast.ncbi.nlm.nih.gov/Blast.cgi) with a cut‐off E‐value of 10^−10^ and the genomic DNA databases of *H. annuus* r1.2 genomes in Phytozome v12.1 (https://phytozome.jgi.doe.gov/pz/portal.html). Gene ontology (GO) was used to achieve annotations including molecular function, cellular component, and biological process ontologies. We further used Kyoto Encyclopedia of Genes and Genomes (KEGG, https://www.kegg.jp/) and EuKaryotic Orthologous Groups (KOG, ftp://ftp.ncbi.nih.gov/pub/COG/KOG/kog) to perform pathways annotation and predict possible functions, respectively.

## 3 RESULTS

### 3.1 Genetic and epigenetic diversity

To guarantee the accuracy of results, we separately performed repeatability test using two independent TD and TMD capillary electrophoresis. The results showed that both TD and TMD had high repeatability (the independent sample *t* test, *p* > 0.05).

For TE genetic variation, we detected 1,230–1,494 loci at the population level, with the percentage of polymorphic loci of 88.72%–99.14%, the Shannon information index (*I*) of 0.27–0.37, and unbiased expected heterozygosity (*uHe*) of 0.18–0.25. At the species level, the total loci reached 1,802. The percentage of polymorphic loci, Shannon information index, and unbiased expected heterozygosity were 100%, 0.40, and 0.25, respectively (Table 2). AMOVA analysis showed that the genetic variation was mainly partitioned within populations (76.14%–88.28%, *p* < 0.001); and variation among populations within regions and among regions were 7.2%–17.24% and 3.42%–11.64% (*p* < 0.001), respectively (Table 3).

**TABLE 2:** Genetic and epigenetic diversity of *Mikania micrantha* based on TEs

TE	Loci		*%P*		*I*		*uHe*		*%Per*	*%Hemi*	*%Non*
Superfamily	TD	TMD	TD	TMD	TD	TMD	TD	TMD	TMD	TMD	TMD
Ty1‐Copia	341	638	100.00	100.00	0.40	0.29	0.25	0.16	41.32	33.02	25.66
Ty3‐Gypsy	376	329	100.00	100.00	0.41	0.29	0.26	0.17	36.31	28.78	34.91
LARD	41	78	100.00	100.00	0.36	0.29	0.22	0.16	49.55	22.25	28.20
TRIM	46	93	100.00	100.00	0.47	0.31	0.31	0.18	41.91	20.52	37.57
LINE	62	166	100.00	100.00	0.39	0.30	0.24	0.17	29.25	39.77	30.98
SINE	128	140	100.00	100.00	0.43	0.26	0.27	0.15	50.59	20.03	29.38
MITE	340	445	100.00	100.00	0.40	0.30	0.25	0.17	26.06	32.04	41.90
MUDR	13	93	100.00	100.00	0.32	0.31	0.19	0.18	30.99	27.98	41.04
Tc1‐Mariner	36	81	100.00	100.00	0.40	0.29	0.25	0.16	36.75	18.86	44.39
Helitron	172	337	100.00	100.00	0.37	0.30	0.23	0.18	43.85	22.68	33.47
CACTA	104	134	100.00	100.00	0.40	0.28	0.25	0.16	56.27	13.06	30.67
PIF‐Harbinger	143	142	100.00	100.00	0.42	0.27	0.27	0.15	46.39	28.78	24.83
All TEs	1,802	2,676	100.00	100.00	0.40	0.29	0.25	0.17	37.97	28.32	33.71

Note

*%P,* percentage of polymorphic loci; *I,* Shannon information index; *uHe,* unbiased expected heterozygosity; *%Per*, percentage of permethylation; *%Hemi*, percentage of hemimethylation; *%Non*, percentage of nonmethylation.

**TABLE 3:** Molecular variance (AMOVA) of *Mikania micrantha* based on TE data

TE superfamily	Among regions		Among populations within regions		Within populations	
	TD	TMD	TD	TMD	TD	TMD
Ty1‐Copia	8.09	2.43	12.87	6.88	79.03	90.69
Ty3‐Gypsy	6.49	3.74	12.15	5.60	81.36	90.66
LARD	5.54	9.77	14.33	10.40	80.13	79.83
TRIM	7.08	6.40	10.59	11.22	82.33	82.38
LINE	3.42	5.66	12.39	8.72	84.20	85.62
SINE	7.07	2.84	10.34	11.66	82.60	85.50
MITE	6.47	3.73	9.73	7.41	83.80	88.86
MUDR	6.62	5.51	17.24	5.84	76.14	88.66
Tc1‐Mariner	4.52	2.12	7.20	5.67	88.28	92.21
Helitron	6.86	4.69	15.03	6.67	78.11	88.64
CACTA	3.76	4.58	12.12	11.97	84.12	83.45
PIF‐Harbinger	11.64	3.28	9.95	7.57	78.40	89.15
All TEs	6.86	3.95	11.83	7.60	81.31	88.45

Note

Values are given in percentage of the total genetic variance and estimated with 10,000 permutations (*p* < 0.001).

Generally, TEs in plants are targeted for methylation and have higher methylation level compared to random genomic sequences (Venetsky et al., 2015). Here we have measured the level of methylation for 306 individuals in 21 populations. In total, we obtained 1,700–2,123 epiloci at the population level. Their percentage of polymorphic epiloci and Shannon information index (*I*) were 97.78%–100% and 0.21–0.29, respectively, while unbiased expected heterozygosity (*uHe*) was 0.13–0.19. At the species level, we acquired 2,676 epiloci with the percentage of polymorphic epiloci of 100%, Shannon information index of 0.29, and unbiased expected heterozygosity of 0.17. The percentage of permethylation (*%Per*), hemimethylation (*%Hemi*), and nonmethylation (*%Non*) were 31.91%–41.54%, 24.80%–30.14%, and 30.30%–38.47%, respectively. Among the 12 TE superfamilies, MITE‐, MUDR‐, and Tc1‐Mariner‐TMD exhibited low level of methylation (Table 2). The epigenetic variation was mainly partitioned within populations (79.83%–92.21%); 5.60%–11.97% was partitioned among populations within regions (*p* < 0.001), and only 2.12%–9.77% was partitioned among regions (*p* < 0.001) (Table 3).

For each TE superfamily, MUDR‐TD presented the highest Shannon information index and unbiased expected heterozygosity, while the lowest values occurred in SINE. For TMD, the highest percentage of permethylation (*%Per*) appeared in CACTA superfamily, while the lowest in MITE. In contrast, CACTA superfamily had the lowest hemimethylation (*%Hemi*), while LINE had the highest. Tc1‐Mariner and PIF‐Harbinger had the highest and lowest nonmethylation (%Non), respectively. Of note, Tc1‐Mariner exhibited the lowest genetic differentiation among populations and regions, while MUDR and PIF‐Harbinger showed the highest genetic differentiation among populations and regions, respectively. As for epigenetic differentiation among populations, Tc1‐Mariner was still the lowest, and LARD was the highest. LARD‐TMD maintained the highest epigenetic differentiation among regions, while Ty1‐Copia and Tc1‐Mariner had the lowest.

Mantel test of TD and TMD revealed that isolation by distance occurred in six TE superfamilies: Ty1‐Copia, Ty3‐Gypsy, LARD, SINE, MITE, and CACTA (*p* < 0.05). In contrast, partial Mantel tests showed that six TEs had a significant pattern of IBD under the control of epigenetic variation, whereas seven TEs yielded a significant pattern of IBD under the control of genetic variation.

Regarding TD, 11 and nine TEs were identified to have bottleneck signatures under the infinite allele model (I.A.M.) and the stepwise mutation model (S.M.M.), respectively (*p* < 0.05) (Table 4). For TMD, only one TE was detected to have bottleneck signatures under I.A.M.

**TABLE 4:** Bottleneck detection for each adopted transposon superfamily

TE markers	I.A.M.		S.M.M.	
	TD	TMD	TD	TMD
Ty1‐Copia	208/133*	251/387	182/159*	184/454*
Ty3‐Gypsy	241/135*	135/194	220/156*	109/220*
LARD	24/17*	29/49	20/21	22/56*
TRIM	37/9*	48/45*	34/12*	36/57
LINE	34/28*	77/89*	31/31	53/113*
SINE	82/46*	44/96	72/54*	32/108*
MITE	218/122*	185/260*	185/155*	146/299*
MUDR	7/6	44/49*	5/8	35/58
Tc1‐Mariner	21/15*	32/49	21/15*	27/54*
Helitron	98/74*	156/181*	84/88*	121/216*
CACTA	61/43*	50/84	56/48*	39/95*
PIF‐Harbinger	98/45*	52/90	87/56*	37/105*
All TEs	1,123/679*	1,108/1,568*	993/809*	835/1,841*

Note

Ratio = He/Hd, heterozygosity excess/deficiency; *p < 0.05.

**FIGURE 2:**
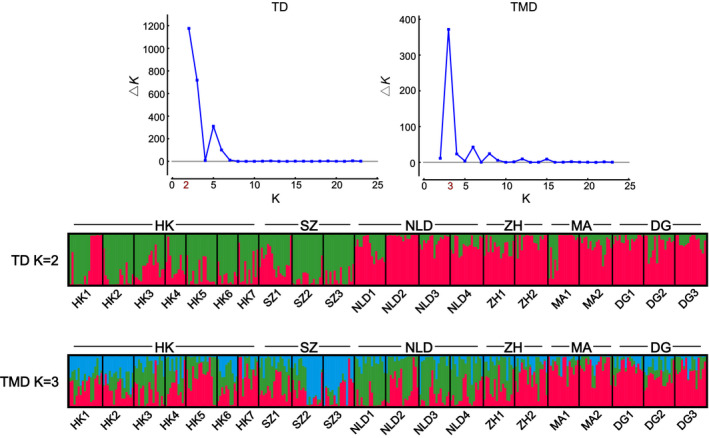
Ty1‐Copia‐based genetic and epigenetic structure of *Mikania micrantha*. The optimal *K* was determined based on the highest Δ*K* value (Evanno et al., 2005). Each bar represents one individual. See Table S1 for population codes

**FIGURE 3:**
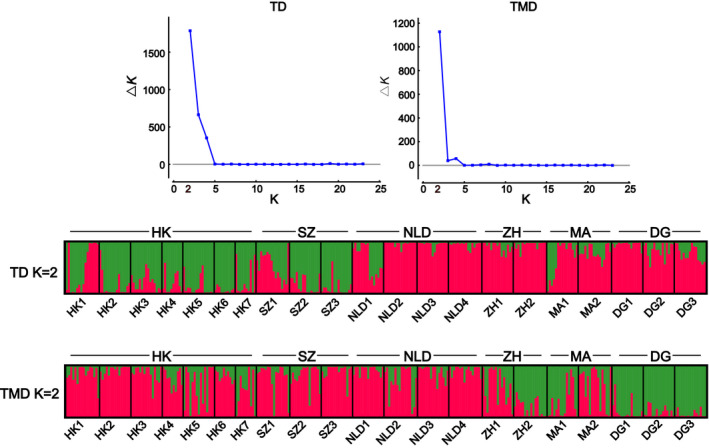
Ty3‐Gypsy‐based genetic and epigenetic structure of *Mikania micrantha*. The optimal *K* was determined based on the highest Δ*K* value (Evanno et al., 2005). Each bar represents one individual. See Table S1 for population codes

Except for Ty1‐Copia and Ty3‐Gypsy superfamilies, TEs exhibited a great gene flow and a mixed structure for *M. micrantha* populations by using TD and TMD. The optimal cluster *K*‐value was selected as two with Structure Harvester. Hong Kong and Shenzhen populations formed cluster I, whereas the other populations comprised cluster II (Figure 2 and Figure 3). PCA obtained similar results except that some individuals from Macao populations were clustered into cluster I when using TD (Figure S1 and Figure S2). There were only two TMD markers showed that *M. micrantha* populations maintained a weak epigenetic structure.

When analyzing spatial genetic structure by TD, only TRIM TEs were found to have a positive Moran *I* value at distance class 0–10 km, and Tc1‐Mariner TEs had a positive Moran *I* value at distance class 10–20 km. By TMD, six TEs were detected to have a positive Moran *I* value at distance class 0–10 km.

When examining fine‐scale genetic structure by TD, all TE superfamilies were detected to have positive kinship coefficients at the intra‐group level. Ten TE superfamilies were found to have positive kinship coefficients at distance class 0–0.15 km, while nine were observed to have positive kinship coefficients at distance class 0.15–0.30 km. Corresponding to the above three levels, all, 11, and three TE superfamilies were identified to have positive kinship coefficients by TMD, respectively. The kinship coefficients were highest at the first level and rapidly decreased.

### 3.2 Candidate loci for environmental adaptation

Due to uneven mutation rates, we performed independent genome scans for each TE superfamily. In total, 145 loci were identified as outliers using both Dfdist and BayeScan, including 59 genetic loci and 86 epigenetic loci (Table 5). Epigenetic outliers were found in all TE superfamilies, while genetic outliers were found in ten. Among them, Ty3‐Gypsy, Ty1‐Copia, and MITE had the most genetic selective loci, while Helitron and Ty1‐Copia possessed the most epigenetic selective loci (Table 5). Of note, all the 145 identified outlier loci were sequenced.

**TABLE 5:** TE‐adaptive loci identified simultaneously by Dfdist and Bayescan

TE	Dfdist		Bayescan		Adaptive loci	
Superfamily	TD	TMD	TD	TMD	TD	TMD
Ty1‐Copia	46	56	23	24	13	12
Ty3‐Gypsy	40	25	25	12	14	7
LARD	3	7	1	6	1	2
TRIM	1	20	0	5	0	4
LINE	6	17	1	9	0	5
SINE	9	23	3	12	2	4
MITE	38	29	21	12	12	4
MUDR	4	8	2	2	2	2
Tc1‐Mariner	0	3	0	2	0	2
Helitron	36	35	7	24	3	15
CACTA	7	16	1	7	1	4
PIF‐Harbinger	12	13	8	4	5	2
All TEs	235	264	98	171	59	86

Eleven TE superfamilies, except MUDER, were found to have genetic loci (109 in total) displaying linkage disequilibrium based on TD data (TE‐TD‐LD), of which PIF‐Harbinger accounted for the largest proportion. By contrast, twelve TE superfamilies all had epiloci (110) displaying linkage disequilibrium based on TMD data (TE‐TMD‐LD); of them, LARD had the highest proportion, while Ty1‐Copia and MITE had the lowest. Percentage of TE‐TD LD to total loci and to adaptive loci ranged from 0% to 1.30% and from 0% to 43.94%, respectively. Percentage of TE‐TMD‐LD to total and adaptive loci was from 0% to 0.67% and 0 to 100%, respectively (Table 6).

**TABLE 6:** Percentage of linkage disequilibrium between pairwise TE‐associated loci

TE markers	*%LD*		*%LDA*	
	TD	TMD	TD	TMD
Ty1‐Copia	0.24	0.05	10.26	7.58
Ty3‐Gypsy	0.15	0.08	15.38	4.76
LARD	0.49	0.67	0.00	100.00
TRIM	0.39	0.65	0.00	100.00
LINE	1.06	0.23	0.00	6.67
SINE	0.76	0.59	0.00	0.00
MITE	0.22	0.05	43.94	0.00
MUDR	0.00	0.19	0.00	100.00
Tc1‐Mariner	0.63	0.15	0.00	0.00
Helitron	1.20	0.12	0.00	3.81
CACTA	1.25	0.22	0.00	83.33
PIF‐Harbinger	1.30	0.15	0.00	0.00
All TEs	0.09	0.10	6.16	3.01

Note

*%LD*, percentage of linkage disequilibrium between total loci; *%LDA*, percentage of linkage disequilibrium between adaptive loci.

**FIGURE 4:**
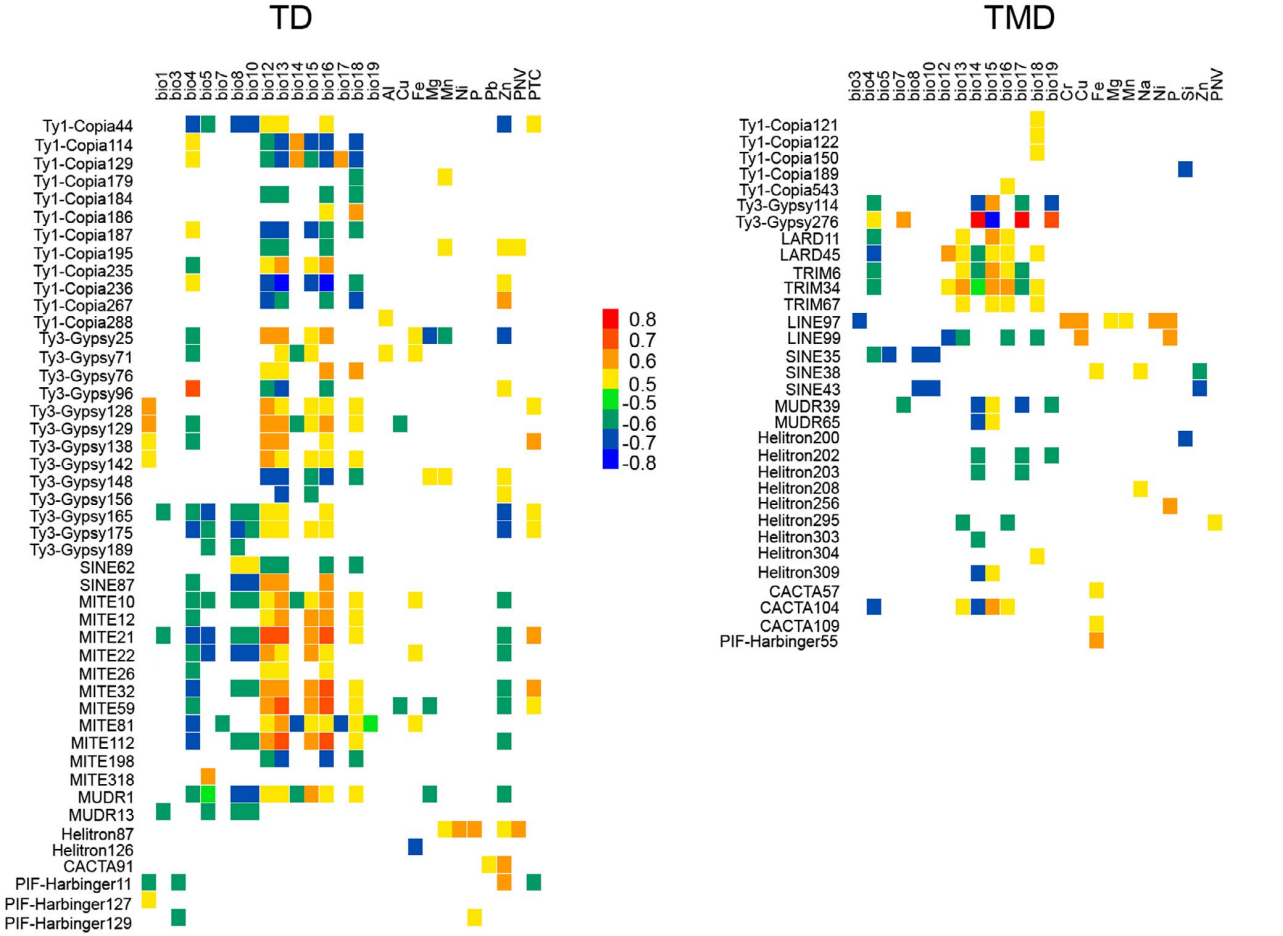
Association of genetic and epigenetic adaptive loci with environment variables. Colors represent correlation values *r* ^2^. Positive and negative values refer to positive and negative correlation, respectively. The greater the absolute value, the stronger the correlation, and vice versa

Using the logistic regression model in Samβada v0.4.5 and the univariate linear regression model in SAM v4.0, we identified 51 genetic loci and 44 epigenetic loci that were correlated with at least one of the environmental variables. Some loci were associated with more than one environmental variable. For genetic loci, there were six loci detected to significantly related to temperature, five to precipitation, seven to metal, and five to vegetation, respectively (*r* ^2^ ≥ 0.5, Figure 4). For epigenetic loci, there were seven, eight, six, and one found to related to the corresponding environmental variables, respectively (Figure 4).

### 3.3 Identification of transposon‐inserted genes

By sequencing of bands extracted from polyacrylamide gel, 29 genetic loci and 77 epigenetic loci were identified as genes or intergenic sequences. Ten TE superfamilies were identified to locate in 25 genes by BLAST analysis (Table 7).

**TABLE 7:** Annotation of transposon‐inserted gene based on BLAST analysis

TE superfamily	Gene	E‐value	Protein
Ty1‐Copia	HanXRQChr09g0262441	4.60E−65	Probable SAM MTase superfamily protein
	HanXRQChr14g0441281	1.70E−116	Putative C2 domain
	HanXRQChr04g0121111	7.10E−43	Probable SKP1‐like 20
Ty3‐Gypsy	HanXRQChr09g0244221	5.40E−28	Putative EGF‐like calcium‐binding domain
	HanXRQChr04g0095781	4.10E−64	Probable ubiquitin‐conjugating enzyme family protein
	HanXRQCPg0580291	3.70E−18	Putative ribosomal protein S12/S23
	HanXRQChr04g0103051	5.10E−11	Probable G2/mitotic‐specific cyclin C13−1
TRIM	HanXRQChr04g0094301	3.30E−52	ATP‐dependent Clp protease proteolytic subunit
SINE	HanXRQChr14g0445931	3.40E−49	Probable splicing factor, CC1‐like
	HanXRQChr09g0263761	2.00E−132	Putative myc‐type, basic helix‐loop‐helix (bHLH) domain
MITE	HanXRQChr14g0441741	2.80E−11	Putative winged helix‐turn‐helix DNA‐binding domain
	HanXRQChr17g0555241	4.00E−46	Putative NB‐ARC
	HanXRQChr02g0058701	2.20E−49	Putative glycolipid transfer protein domain
	HanXRQChr09g0267451	2.90E−114	Probable RING/U‐box superfamily protein
	HanXRQChr02g0051731	9.00E−20	Probable (‐)‐beta‐pinene synthase, chloroplastic
	HanXRQChr05g0145441	2.50E−48	Probable cryptochrome 1
	HanXRQChr12g0373791	2.10E−55	Probable subtilisin‐like serine endopeptidase family protein
MUDR	HanXRQChr15g0470721	3.50E−56	Probable 5'‐adenylylsulfate reductase 1, chloroplastic
Tc1‐Mariner	HanXRQChr05g0149891	1.00E−57	Putative peptidase C1A
Helitron	HanXRQChr09g0272091	1.60E−32	Putative WD40/YVTN repeat‐like‐containing domain
	HanXRQChr09g0253961	5.40E−68	Probable ribonucleotide reductase 1
CACTA	HanXRQChr02g0052361	3.00E−19	Auxin response factor
	HanXRQChr16g0500791	2.70E−52	Putative proton‐dependent oligopeptide transporter family
PIF‐Harbinger	HanXRQChr05g0157761	2.50E−118	Probable SWAP/surp domain‐containing protein
	HanXRQChr09g0272861	3.80E−98	Probable galactose oxidase/kelch repeat superfamily protein

**FIGURE 5:**
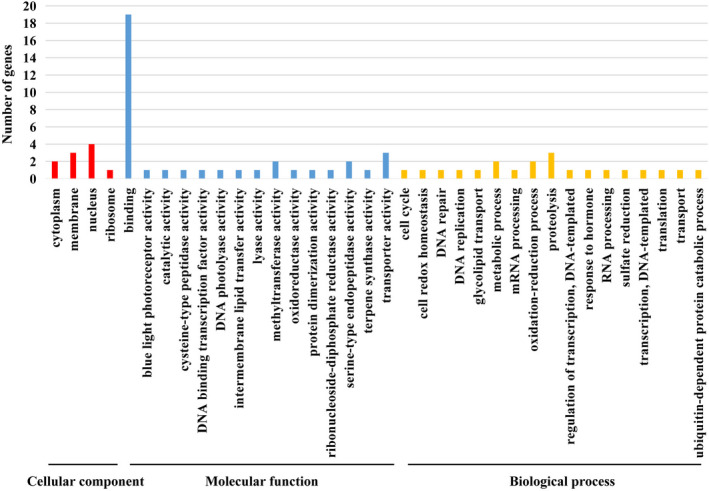
Gene ontology assignment of transposon‐inserted genes in terms of cellular component (Red), molecular function (Blue), and biological process (Orange)

**FIGURE 6:**
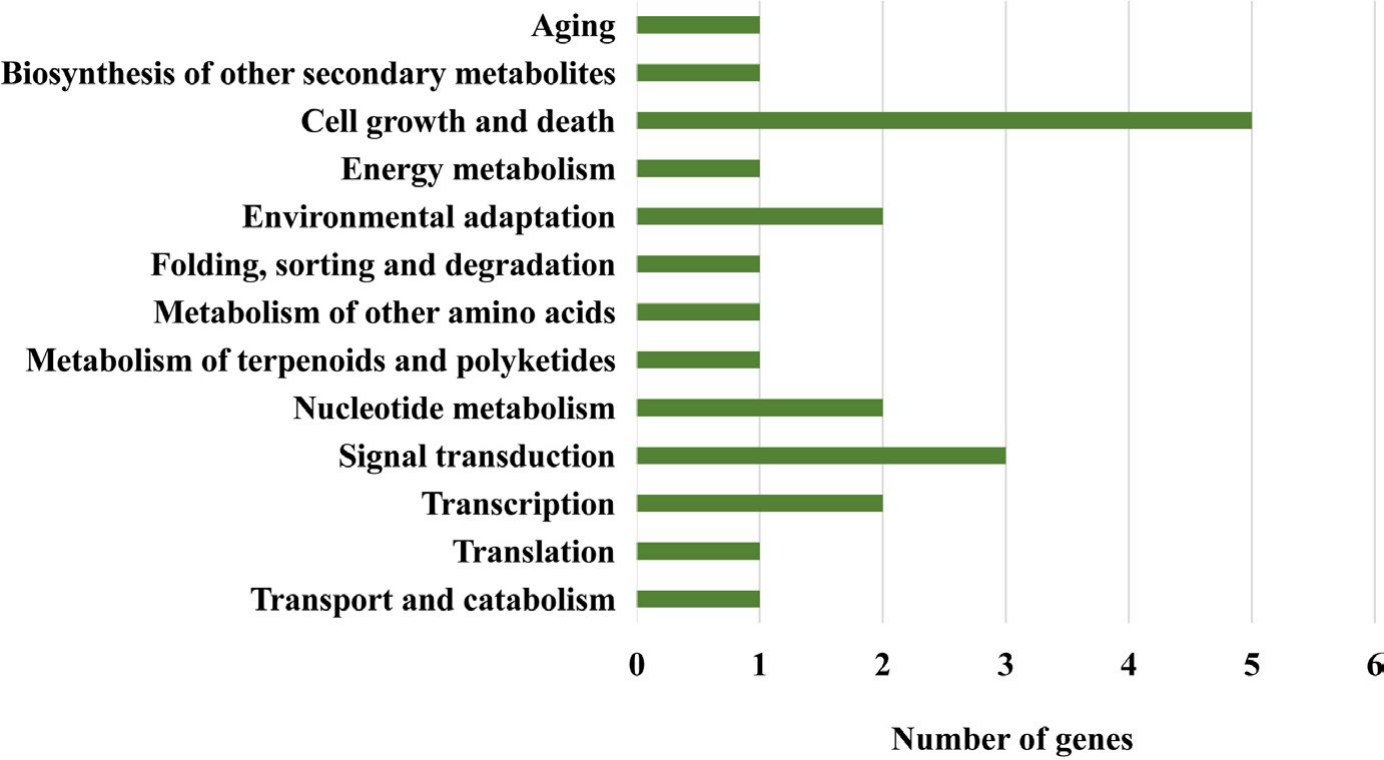
Kyoto Encyclopedia of Genes and Genomes (KEGG) pathway annotations of transposon‐inserted genes showing they are involved in cell growth and death, signal transduction, and environmental adaption. The listed are the specific pathways classified under KEGG categories

The 25 genes were further annotated by Gene Ontology (GO), Kyoto Encyclopedia of Genes and Genomes (KEGG) pathway, and EuKaryotic Orthologous Groups (KOG). Based on GO terms, they were assigned to 36 categories, including four cellular components, 15 molecular functions, and 17 biological processes (Figure 5). Of them, genes related to binding were mostly enriched (Figure 5). KEGG pathway analysis showed that 12 genes were attributed to 13 biological pathways, such as cell growth and death, signal transduction, and environmental adaption (Figure 6). Moreover, 16 genes were assigned to nine KOG categories, most of which were involved in post‐translational modification/protein turnover/chaperone functions, amino acid metabolism and transport, and RNA processing and modification (Figure S3).

The annotation results indicated that Ty1‐Copia and Ty3‐Gypsy separately inserted into genes related to probable S‐adenosyl‐L‐methionine‐dependent methyltransferase (SAM Mtase) superfamily protein and ubiquitin‐conjugating enzyme family protein, genes related to MITE insertion were probable (−)‐β‐pinene synthase and probable cryptochrome 1, and genes with insertion of Helitron, CACTA, and PIF‐Harbinger were associated with probable ribonucleotide reductase 1, auxin response factor, and kelch repeat superfamily protein, respectively (Table 7).

## 4 DISCUSSION

### 4.1 (Epi) genetic diversity of transposable elements

Since it is hard to examine all TEs, representative candidate TEs were selected in this study, which focuses on high‐copy‐number families, low‐copy‐number LTR retrotransposons, and other TEs such as MITEs, LINEs, and SINEs. Their genetic and epigenetic variation have been detected by using the method of TD and TMD in *M. micrantha*, and the relative contribution of genetic and epigenetic variation to genetic differentiation has also been dissected. Generally, genomes of Asteraceae plants have been observed to have abundant transposons, which can cause genome size variation and impose influence on growth and development in multiple aspects (Giordani et al., 2014). As a noxious invasive plant in southern China, *M. micrantha* offers an opportunity to pursue the solution of the well‐known paradox in invasion biology: invasion success despite the occurrence of initial or repeated demographic and genetic bottlenecks (Estoup et al., 2016; Frankham, 2005; Geng et al., 2017; Schrieber & Lachmuth, 2017; Wang et al., 2008, 2012). Transposable elements might help explain this phenomenon as they enable the generation of a great variety of mutations in the plant genome, which might counteract genetic depletion and thus allow for a quick response to novel environments (Casacuberta & González, 2013). The present study observed considerable genetic and epigenetic variation of TEs in *M. micrantha*. Compared to genetic variation based on other molecular markers like ISSR (Wang et al., 2008) and AFLP (Wang et al., 2012), the genetic variation of transposons is relatively high as shown by the percentage of polymorphic loci and Shannon information index but not by the unbiased expected heterozygosity (Table 8). Similarly, high levels of epigenetic variation of transposons were observed as well (Table 8) compared to results of MSAP (Methylation‐Sensitive Amplification Polymorphism; Shen et al., 2021). The relatively high levels of TE genetic and epigenetic variation reflect that TEs are quite active in *M. micrantha* genome and contribute to enhancing variability (Alleman & Freeling, 1986; Tam et al., 2009). This might bring evolutionary raw material for the weed to withstand environmental changes and stresses, although a significant bottleneck effect was detected in all the TE superfamilies analyzed except MUDER. Our results highlight the potential importance of TE genetic and epigenetic variation for the rapid adaptation and invasion of *M. micrantha*. Richards et al. (2012) have suggested that epigenentic effects could contribute to phenotypic variation in genetically depauerate invasive populations of Japanese kotweed (*Fallopia* species complex) by performing AFLP and MSAP analysis. Following this line, it will be of considerable interest to characterize the association between TE epigenetic variation and phenotypic variation in the *M. micrantha* populations.

**TABLE 8:** Comparison of genetic and epigenetic parameters of *Mikania micrantha* between TE‐associated and other markers

Markers		*%P*	*I*	*uHe*	*%Per*	*%Hemi*	*%Non*
ISSR	Population	25.90−59.66%	0.11–0.25	0.09–0.21			
Wang et al. (2008)	Region	57.31−75.39%	0.20–0.25	0.19–0.22			
	Species	85.65%	0.25	0.24			
AFLP	Population	10.57−73.47%		0.04–0.29			
Wang et al. (2012)	Region	52.62−93.25%		0.20–0.33			
	Species	100%		0.34			
TD	Population	88.72−99.14%	0.27–0.37	0.18–0.25			
This study	Region	97.59−100%	0.34–0.39	0.22–0.25			
	Species	100%	0.40	0.25			
MSAP Shen et al. (2021)	Population	31.10−41.80%	0.11–0.14	0.07–0.09	19.40−46.89%	33.89−68.33%	12.28−26.11%
Unpublished	Region	41.90−78.60%	0.12–0.15	0.07–0.09	22.11−46.40%	34.23−62.85%	15.04−20.25%
	Species	100%	0.15	0.08	39.09%	41.69%	19.23%
TMD	Population	97.98−100%	0.21–0.29	0.13–0.19	30.44−44.67%	20.58−34.05%	27.95−41.19%
This study	Region	99.92−100%	0.23–0.30	0.13–0.18	31.91−41.54%	24.80−30.14%	30.30−38.47%
	Species	100%	0.29	0.17	37.97%	28.32%	33.71%

Note

*%P*, percentage of polymorphic loci; I, Shannon information index; *uHe*, unbiased expected heterozygosity; *%Per,* percentage of permethylation; *%Hemi*, percentage of hemimethylation; *%Non*, percentage of nonmethylation.

Moreover, epigenetic variation is generally assumed to be greater than genetic variation in plants due to the frequent occurrence of retrotransposons (Roy et al., 2015). In accordance with this, by using AFLP and MSAP markers Shi et al. (2019) detected an extremely low genetic diversity but nearly 20‐fold higher epigenetic diversity in the invasive Chinese populations of Alligator weed (*Alternanthera philoxeroides*); Liu et al. (2018) observed a higher value of diversity indices for epigenetic than genetic diversity in the invasive North America populations of common reed (*Phragmites australis*). And similar finding was noted for Japanese kotweed too. Nevertheless, nearly the same level of both types of TE variations was detected in *M. micrantha*, which deserves further study.

### 4.2 (Epi) genetic structure and spatial (epi) genetic structure

Transposon variation in plant genome may be more suitable to estimate population structure in comparison to other molecular markers (Bonchev & Parisod, 2013; Kidwell & Lisch, 1997). In this study, a mixed structure was obtained in the populations of *M. micrantha* by using other TEs except for Ty1‐Copia and Ty3‐Gypsy. It is of note that when conducting the analysis based on Ty1‐Copia/Ty3‐Gypsy data, a structure was detected: Hongkong and Shenzhen populations formed a cluster, and the other regions comprised the other (Figure 2 and Figure 3). This pattern mirrors the geographical proximity of Hongkong and Shenzhen. Ty1‐Copia and Ty3‐Gypsy are retrotransposons whose semi‐conservative “copy‐and‐paste” mechanism may impose an effect on their variation (e.g., genome expansion and high mutation rate), making them more informative for characterizing population (epi) genetic differentiation (Kalendar et al., 2011; Kidwell & Lisch, 1997; Roy et al., 2015). In combination with previous ISSR and AFLP results (Wang et al., 2008, 2012), we suggest that invasive populations of *M. micrantha* in southern China have been differentiated genetically and epigenetically.

Next, broad‐scale and fine‐scale spatial genetic structure (BSGS and FSGS) analysis was conducted. For BSGS, a difference was observed between the positive spatial correlation patterns established from TD or TMD data. Namely, a positive correlation was revealed at both class 0–10 and 10–20 km when using TD, whereas a positive correlation can only be found at class 0–10 km using TMD. For FSGS, by contrast, a similar pattern was uncovered based on TD or TMD data. A positive correlation was identified at levels of intra‐group, 0–0.15, and 0.15–0.30 km by using either method. A similar positive spatial (epi) genetic structure pattern was also observed within 300 m. Causal factors that contribute to the spatial (epi) genetic structure of *M. micrantha* possibly include asexual reproduction, light, and small seeds with pappus, and dispersal of propagules by wind, water, and animal vectors (Guo et al., 2005).

Moreover, significant genetic and epigenetic IBD patterns were observed. This is in accordance with the findings by using MSAP (Shen et al., 2021) but not by using AFLP (Wang et al., 2012), underscoring the effects of molecular marker choice on the pattern estimation.

### 4.3 Adaptive loci associated with TEs

The roles of TEs have previously been documented for the adaptation of invasive plants to novel environments (Gonzalez et al., 2010; Goubert et al., 2017; Goubert et al., 2017; Stapley et al., 2015). This study identified more epigenetic (86) than genetic (59) candidate adaptive loci by applying genome scans. Epigenetic adaptive loci also appear in more TE superfamilies (100%) than genetic ones (91.7%) (Table 5). Overall linkage disequilibrium is higher in genetic and epigenetic adaptive loci than in nonadaptive loci (Table 6), which also suggests the presence of selection on adaptive loci (Vasemagi et al., 2005).

Importantly, both epigenetic and genetic adaptive loci have been observed significantly correlated with such environmental factors as temperature, precipitation, soil metals, and vegetation (Figure 4). Since being introduced from its tropical native area to southern China, *M. micrantha* has been faced with multiple environmental changes. It has previously been shown that TEs are associated with environmental adaptation (Gonzalez et al., 2010; Magalhaes et al., 2007). Our results further consolidate the roles of TEs, particularly the epigenetic status of TEs, in the invasion of *M. micrantha*.

### 4.4 Genes inserted by TEs

Consequences generated by TE insertion into functional genes include loss of gene function, regulatory change, buildup of epigenetic variation, and induction of novel phenotypes (Bonchev & Parisod, 2013; Schrader & Schmitz, 2019). The affected genes or genomic DNA can be captured by sequencing the excised gel bands and conducting homology‐based annotation. In this work, 29 and 77 bands have been recovered and sequenced in the TD and TMD analysis for *M. micrantha*, respectively. The TEs were found mainly located within coding regions excluding 5′‐ and 3′‐untranslated region (UTR) and introns and intergenic sequences. In total, we identified and annotated 25 genes with TE insertions (Table 7).

The 25 genes targeted by TEs are involved in a variety of molecular and cellular functions. The followings are of particular interest: (a) A gene probably coding SAM‐Mtase, a core enzyme in phenylpropanoid and flavonoid metabolism, is inserted with Ty1‐Copia. The gene may function in plant disease resistance and development (Joshi & Chiang, 1998). (b) A gene putatively coding ubiquitin‐conjugating enzyme is inserted by Ty3‐Gypsy, which is also critical for plant disease resistance (Liu et al., 2016). (c) MITE inserts into a probable *β*‐pinene synthetase gene and a cryptochrome‐encoding gene. Products of the former have a link to allelopathy (Ji et al., 2012; Wang & Zhu, 1996), and the latter is related to light morphogenesis and flowering time (Kleine et al., 2007; Liu et al., 2011). Noteworthily, genes with MITE insertions are also associated with plant immune and stress responses (Table 7; Fontanini & Jones, 2002; Van Ooijen et al., 2008). (d) Helitron inserts into a gene probably coding ribonucleotide reductase 1, which is involved in DNA replication and DNA damage response (Dyavaiah et al., 2011). (e) CACTA inserts into an auxin response factor gene. The factor contributes to regulate DNA replication and to respond to plant hormones. And (f) PIF‐Harbinger inserts into a gene encoding kelch repeat superfamily protein, which functions in the iron deficiency stress response in roots (Kawahara et al., 2017). These results provide new perspectives to understand the underlying mechanism of *M. micrantha* invasion. Meanwhile, they highlight the importance of TE‐associated genetic and epigenetic variation in adaptation.

## 5 CONCLUSION

This study sheds light on the genetic paradox of invasive weed *M. micrantha* based on TD and TMD analysis and has demonstrated that TEs indeed play a key role in its invasion and adaptive evolution. TEs are capable to provide high levels of genetic and epigenetic variation for *M. micrantha* as required for adaptation to novel environments. In combination with the invasive phenotypic characteristics, our results indicate that TEs may affect the rapid adaptation of *M. micrantha* by altering the following aspects: genetic and epigenetic variation, population structure, joint action of adaptive loci with environmental factors, and the methylation status of functional genes.

## CONFLICT OF INTEREST

The authors declare no conflicts of interest.

## AUTHOR CONTRIBUTIONS

**Yingjuan Su:** Formal analysis (equal); Supervision (equal). **Qiqi Huang:** Investigation (equal). **Zhen Wang:** Data curation (equal); Writing‐review & editing (equal). **Ting Wang:** Funding acquisition (equal); Supervision (equal).

## Supporting information

Figure S1

Figure S2

Figure S3

Appendix S1‐S2

## Data Availability

The datasets used for this study are available through Dryad at the time of publication (https://doi.org/10.5061/dryad.02v6wwq3v).
