# Supplementary figures and images for: High genetic and epigenetic variation of transposable elements: Potential drivers to rapid adaptive evolution for the noxious invasive weed Mikania micrantha
Source: Ecol Evol. 2021 Sep 15;11(19):13501–17. doi: 10.1002/ece3.8075 (PMC8495827; doi:10.1002/ece3.8075)

# TD

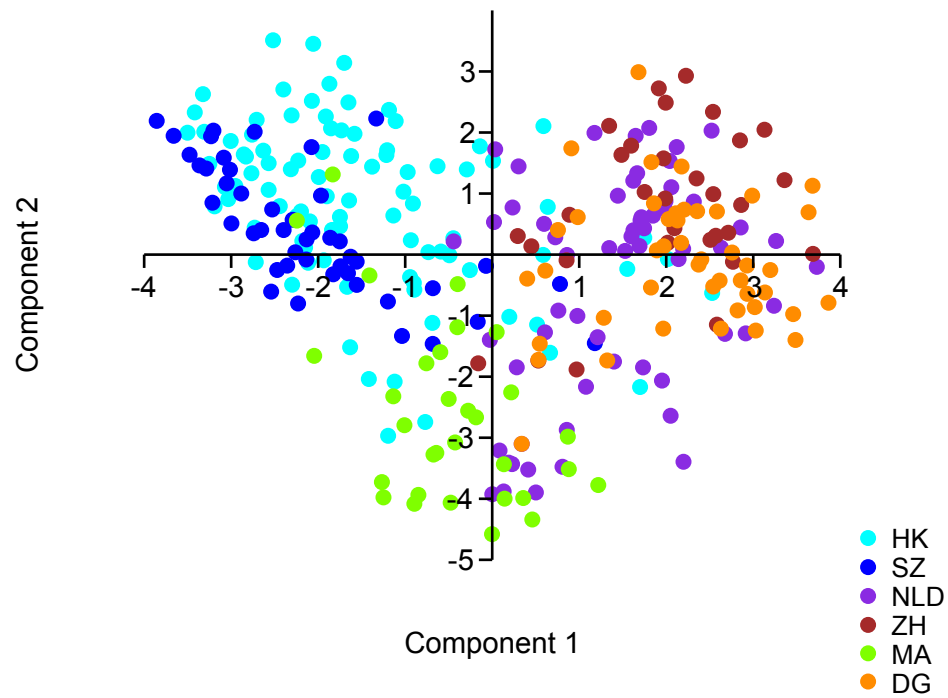

# TMD

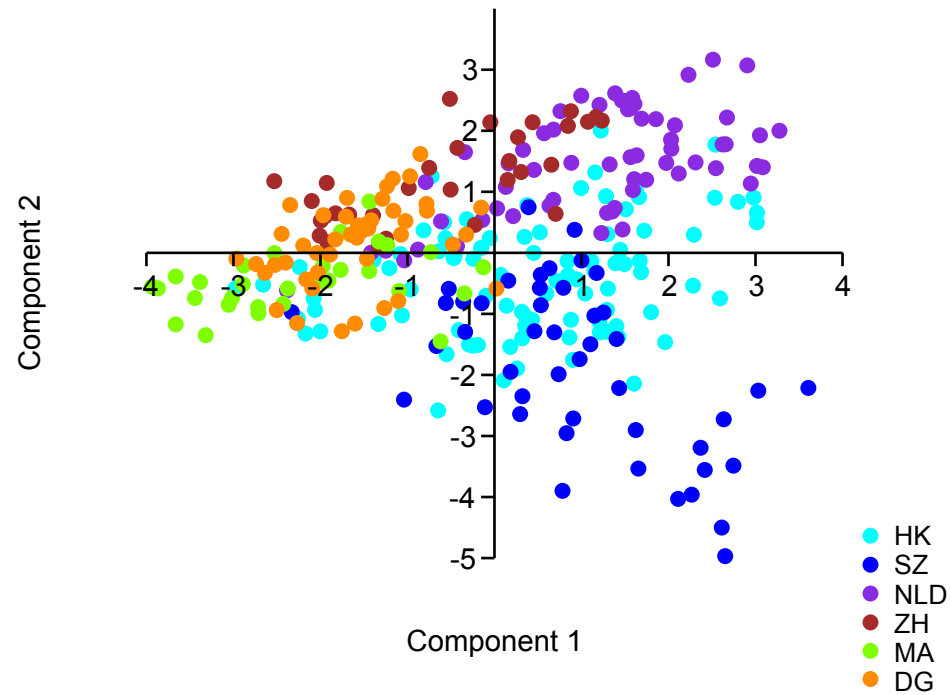

Supplement: Supplementary file 1 — Figure S1 [file ECE3-11-13501-s004.pdf]

# TD

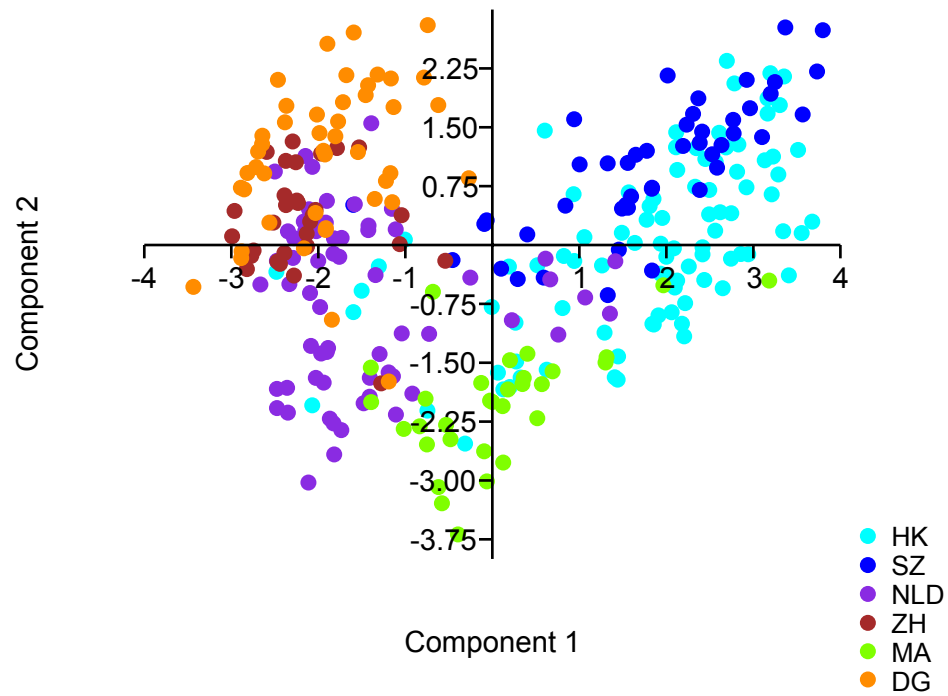

# TMD

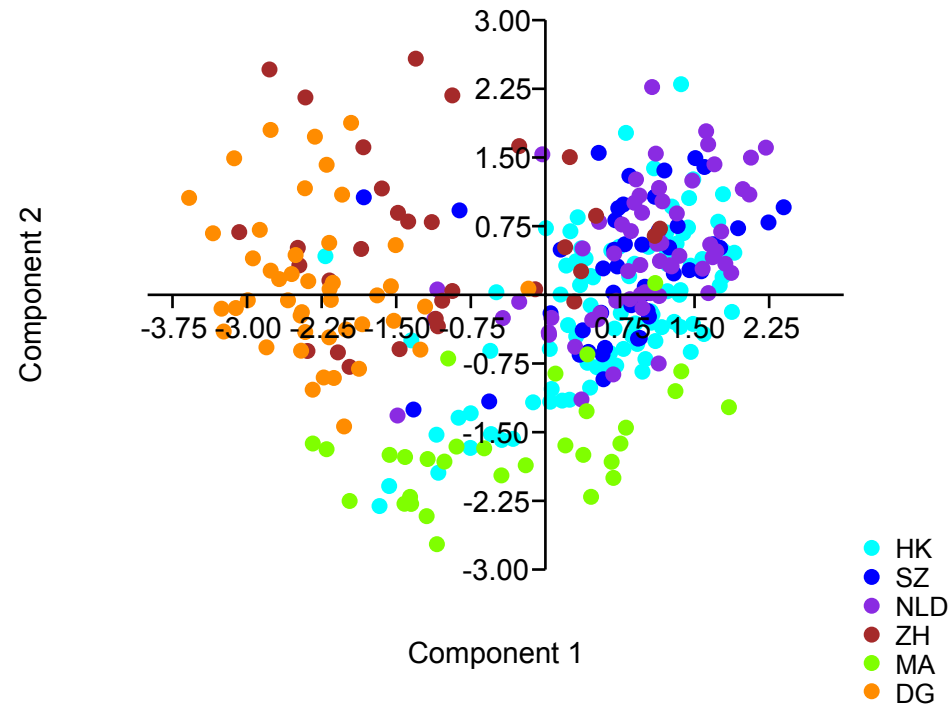

Supplement: Supplementary file 2 — Figure S2 [file ECE3-11-13501-s002.pdf]

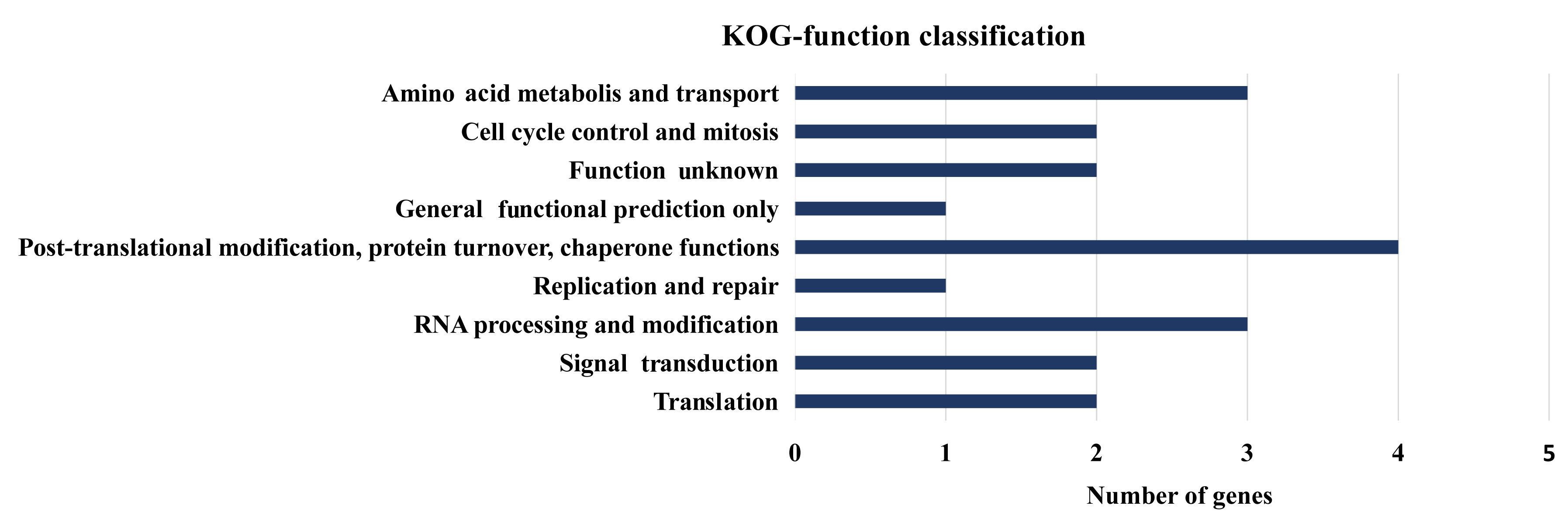

Supplement: Supplementary file 3 — Figure S3 [file ECE3-11-13501-s001.jpg]
