# Supplementary material for: High genetic and epigenetic variation of transposable elements: Potential drivers to rapid adaptive evolution for the noxious invasive weed Mikania micrantha
Source: Ecol Evol. 2021 Sep 15;11(19):13501–17. doi: 10.1002/ece3.8075 (PMC8495827; doi:10.1002/ece3.8075)
Supplement: Supplementary file 4 — Appendix S1‐S2 [file ECE3-11-13501-s003.docx]

Supplementary Material

Appendix 1:

Table S1 | Information of each population for *Mikania micrantha*.

| **Region** | **Population** | **Longitude** | **Latitude** | **Altitude/m.a.s.l** | **Sample size** |
| --- | --- | --- | --- | --- | --- |
| Hong Kong | HK1 | 114°02'33"E | 22°29'22"N | 4 | 16 |
|  | HK2 | 114°14'03"E | 22°23'26"N | 80 | 15 |
|  | HK3 | 114°15'38"E | 22°31'37"N | 4 | 15 |
|  | HK4 | 114°09'10"E | 22°23'49"N | 227 | 10 |
|  | HK5 | 114°08'03"E | 22°23'12"N | 100 | 15 |
|  | HK6 | 114°08'30"E | 22°27'02"N | 39 | 10 |
|  | HK7 | 114°11'49"E | 22°15'25"N | 48 | 10 |
| Shenzhen | SZ1 | 114°03'29"E | 22°33'13"N | 47 | 16 |
|  | SZ2 | 114°02'13"E | 22°34'27"N | 39 | 15 |
|  | SZ3 | 114°10'11"E | 22°34'48"N | 85 | 15 |
| Neilingding | NLD1 | 113°49'01"E | 22°24'32"N | 48 | 15 |
|  | NLD2 | 113°48'14"E | 22°24'13"N | 11 | 16 |
|  | NLD3 | 113°48'00"E | 22°25'14"N | 2 | 15 |
|  | NLD4 | 113°48'43"E | 22°24'56"N | 24 | 16 |
| Zhuhai | ZH1 | 113°37'51"E | 22°25'42"N | 6 | 15 |
|  | ZH2 | 113°38'15"E | 22°24'51"N | 13 | 16 |
| Macao | MA1 | 113°33'20"E | 22°08'25"N | 10 | 15 |
|  | MA2 | 113°33'48"E | 22°07'19"N | 135 | 16 |
| Dongguan | DG1 | 113°48'00"E | 22°52'46"N | 48 | 15 |
|  | DG2 | 113°46'05"E | 22°54'17"N | 182 | 15 |
|  | DG3 | 113°47'34"E | 22°51'15"N | 113 | 15 |

Table S2 | Pre- and selective- amplification primer and sequence originated from Roy et al. (2015)

| Type | Primer name | Primer Sequence |  |
| --- | --- | --- | --- |
| Pre-amplification primer | EcoRI | GACTGCGTACCAATTC |  |
|  | MseI | GACGATGAGTCCTGAGTAA |  |
|  | HpaII/MspI | ATCATGAGTCCTGCTCGG |  |
| Selective amplification primer | Mse-1 | GACGATGAGTCCTGAGTAACTA |  |
|  | Mse-2 | GACGATGAGTCCTGAGTAACAT |  |
|  | Mse-3 | GACGATGAGTCCTGAGTAAGTG |  |
|  | HM-1 | ATCATGAGTCCTGCTCGGCAT |  |
|  | HM-2 | ATCATGAGTCCTGCTCGGTCT |  |
|  | HM-3 | ATCATGAGTCCTGCTCGGGCA |  |
|  | HM-4 | ATCATGAGTCCTGCTCGGAAG |  |

Table S3 | List of climate variables.

| TANN | Annual Mean Temperature |
| --- | --- |
| TMDR | Mean Diurnal Range |
| Iso | Isothermality |
| TS | Temperature Seasonality |
| TMXWM | Max Temperature of Warmest Month |
| TMNCM | Min Temperature of Coldest Month |
| TAR | Temperature Annual Range |
| TWETQ | Mean Temperature of Wettest Quarter |
| TDRYQ | Mean Temperature of Driest Quarter |
| TWMQ | Mean Temperature of Warmest Quarter |
| TCLQ | Mean Temperature of Coldest Quarter |
| PANN | Annual Precipitation |
| PWETM | Precipitation of Wettest Month |
| PDRYM | Precipitation of Driest Month |
| PS | Precipitation Seasonality |
| PWETQ | Precipitation of Wettest Quarter |
| PDRYQ | Precipitation of Driest Quarter |
| PWMQ | Precipitation of Warmest Quarter |
| PCLQ | Precipitation of Coldest Quarter |

Table S4 | List of soil factors.

| Water content | The fresh and dried water content |
| --- | --- |
| pH | pH value of soil suspension |
| Conductivity | Conductivity of soil suspension |
| Organic Matte | Content of organic matte |
| C | Content of total carbon |
| N | Content of total nitrogen |
| P | Content of total phosphorus |
| S | Content of total sulfur |
| Si | Content of total silicon |
| K | Content of kalium |
| Ca | Content of calcium |
| Na | Content of sodium |
| Mg | Content of magnesium |
| Al | Content of aluminum |
| Fe | Content of ferrum |
| Mn | Content of manganese |
| Zn | Content of zinc |
| Cu | Content of cuprum |
| Pb | Content of plumbum |
| Cr | Content of chromium |
| As | Content of arsenic |
| Se | Content of selenium |
| Ni | Content of nickel |
| Cd | Content of cadmium |

Appendix 2:

Ten ecological variables were acquired from the moderate resolution imaging spectroradiometer (MODIS) dataset stored in the Land Process Distributed Active Archive Center (LPDAAC, http://lpdaac.usgs.gov). Normalized difference vegetation index (NDVI) and enhanced vegetation index (EVI) were download from the MOD13A2 dataset (1000 m resolution). Leaf area index (LAI) and fraction of absorbed photosynthetically active radiation (fPAR) were available from the MOD15A2H dataset (500 m resolution). Evapotranspiration (ET) was derived from the MOD16A2 dataset (500 m resolution). Gross primary production (GPP) and photosynthesis (PSN) were obtained from the MOD17A2 dataset (1000 m resolution). Percent of tree cover (PTC), percent of non-tree vegetation cover (PNT), and percent of non-vegetation cover (PNV) were acquired from the MOD44B dataset (250 m resolution). All MODIS datasets corresponded to the years 2006–2016. The yearly mean value for each ecological variable was computed using the maximum value composite function (Huete, Didan, Miura, Rodriguez, Gao, & Ferreira, 2002).

**Reference**

Huete, A., Didan, K., Miura, T., Rodriguez, E. P., Gao, X., & Ferreira, L. G. (2002). Overview of the radiometric and biophysical performance of the MODIS vegetation indices. Remote Sensing of Environment, 83, 195–213.

Appendix 3:

Captions for supplementary figures S1-S3

FIGURE S1 | The PCA (Principal component analysis) plot of *Mikania micrantha* populations by using Ty1-Copia-based TD (Transposon display) and TMD (Transposon methylation display) data, respectively. See Table S1 for population codes.

FIGURE S2 | The PCA (Principal component analysis) plot of *Mikania micrantha* populations by using Ty3- Gypsy-based TD (Transposon display) and TMD (Transposon methylation display) data, respectively. See Table S1 for population codes.

FIGURE S3 | Eukaryotic ortholog group (KOG) function classification of transposon-inserted genes.
